# Parental Report via a Mobile App in the Context of Early Language Trajectories: StarWords Study Protocol

**DOI:** 10.3390/ijerph19053067

**Published:** 2022-03-05

**Authors:** Karolina Mieszkowska, Grzegorz Krajewski, Krzysztof Sobota, Agnieszka Dynak, Joanna Kolak, Magdalena Krysztofiak, Barbara Łukomska, Magdalena Łuniewska, Nina Gram Garmann, Pernille Hansen, Anna Sara Hexeberg Romøren, Hanne Gram Simonsen, Katie Alcock, Napoleon Katsos, Ewa Haman

**Affiliations:** 1 Faculty of Psychology, University of Warsaw, 00-183 Warsaw, Poland; g.krajewski@psych.uw.edu.pl (G.K.); krzysztof.sobotakrk@gmail.com (K.S.); agnieszka.dynak@psych.uw.edu.pl (A.D.); j.kolak@salford.ac.uk (J.K.); magdalena.krysztofiak@psych.uw.edu.pl (M.K.); barbara.lukomska@uw.edu.pl (B.Ł.); magdalena.luniewska@psych.uw.edu.pl (M.Ł.); 2 School of Health & Society, University of Salford, Salford M6 6PU, UK; 3 Department of Early Childhood Education, Faculty of Education and International Studies, Oslo Metropolitan University, 0176 Oslo, Norway; nigrag@oslomet.no (N.G.G.); romo@oslomet.no (A.S.H.R.); 4 MultiLing, Department of Linguistics and Scandinavian Studies, University of Oslo, 0317 Oslo, Norway; pernille.hansen@inn.no (P.H.); h.g.simonsen@iln.uio.no (H.G.S.); 5 Department of Humanities, Faculty of Education, Inland Norway University of Applied Sciences, 2318 Hamar, Norway; 6 Department of Psychology, Lancaster University, Lancaster LA1 4YF, UK; k.j.alcock@lancaster.ac.uk; 7 Faculty of Modern and Medieval Languages and Linguistics, University of Cambridge, Cambridge CB3 9DP, UK; nk248@cam.ac.uk

**Keywords:** language development, bilingualism, vocabulary development, language milestones

## Abstract

Social sciences researchers emphasize that new technologies can overcome the limitations of small and homogenous samples. In research on early language development, which often uses parental reports, taking the testing online might be particularly compelling. Due to logistical limitations, previous studies on bilingual children have explored the language development trajectories in general (e.g., by including few and largely set apart timepoints), or focused on small, homogeneous samples. The present study protocol presents a new, on-going study which uses new technologies to collect longitudinal data continuously from parents of multilingual, bilingual, and monolingual children. Our primary aim is to establish the developmental trajectories in Polish-British English and Polish-Norwegian bilingual children and Polish monolingual children aged 0–3 years with the use of mobile and web-based applications. These tools allow parents to report their children’s language development as it progresses, and allow us to characterize children’s performance in each language (the age of reaching particular language milestones). The project’s novelty rests on its use of mobile technologies to characterize the bilingual and monolingual developmental trajectory from the very first words to broader vocabulary and multiword combinations.

## 1. Introduction

Social sciences research has been inclining towards computer- and web-based testing for more than a decade now, emphasizing that new technologies can overcome the long-standing limitations of small and homogenous samples [1,2,3,4]. The outbreak of the COVID-19 pandemic only heightened the need for online tools for app-based interventions into mental health [5], primary care [6] and, importantly, online testing (e.g., the Cross-linguistic Lexical Tasks app or Naming and Inductive Reference app developed by the Multilingual Language Development and Assessment Lab (MultiLADA) at the University of Warsaw, or the Touchscreen Task for Toddlers developed at Oxford University BabyLab). This is particularly visible in developmental research since access to child participants during the pandemic was radically limited in various countries from the beginning of the pandemic [7,8]. This was one of the motivations for designing the StarWords project and basing it on mobile and web-based technologies. 

Early language development of monolingual and bilingual children is assumed to go through the same milestones at roughly the same time points [9,10]. However, so far, studies have explored the issue in general (e.g., by including 6-month intervals, e.g., [11]) and have not managed to explore the early bilingual language trajectory from the very first words to a larger vocabulary size (e.g., that of 50 words). This is partly due to the logistic difficulties connected with such studies, both in monolingual and bilingual settings: if they collected language samples, recording sessions then needed to be transcribed and analyzed, which significantly lengthened the studies. In a seminal study of Hart & Risley [12], the authors visited 42 families once a month (for two and a half years) to record an hour of the children’s casual interactions with the parents. This amounted to over 1300 h of recordings, and 6 years of data transcription, coding and analysis [13]. Even in a more recent attempt to study early language trajectory, with more advanced technology, Roy et al. [14] have undertaken a three-year-long recording session, leading to 90,000 h of video recording and 140,000 h of audio recording, all collected from one family. Studies that did not collect audio data from the participants, but asked the parents to monitor or report on their children’s early vocabulary, also usually included small sample sizes (e.g., [15]) or samples with distinctive characteristics, e.g., coming from Canada where the two languages enjoy an equal status (see e.g., [16] for further explanation). Previous studies also controlled for few factors [17,18] and did not minutely time the children’s attainment of particular language milestones.

The available evidence for similar developmental trajectories between bilingual and monolingual children comes with some reservations: this result is only obtained when the two languages of the bilinguals are considered (e.g., total vocabulary, summed across two languages). In fact, in single language development, bilinguals may trail behind monolinguals in reaching early language milestones [11]. This may be linked to the bilingual-monolingual differences in language input. The limited exposure to a single language in bilinguals leads to reduced vocabulary size in that language as compared to monolinguals [11]. Furthermore, the amount of exposure to each language may change over the years and across contexts (e.g., home vs. educational settings). As a consequence, the bilingual’s fluency in each language is not fixed, but subject to exposure factors that may fluctuate over the years, reflecting the changes in the immediate linguistic environment [19,20]. For now, there is a lack of longitudinal studies of larger groups of children which scrupulously compare bilingual and monolingual developmental paths (and bilingual development in both languages). Further, there is a need for investigations that minutely time the children’s reaching of early language milestones (e.g., production of first words and multiword utterances). 

Thus, current studies on early language trajectories in bilingual children, with the purpose of establishing the developmental trajectory in bilingual children and providing evidence for early screening practice and accurate identification of potential language delays, should meet a certain set of criteria. First of all, they should follow a large sample longitudinally. The sample should also be heterogeneous, i.e., representative of the population and including participants that differ in terms of their characteristics, e.g., socio-economic status, patterns of language use in the family, children’s age of exposure to each language, and parental knowledge of the majority language. Second, the studies should include multiple measurement points or even use continuous data collection methods. Last, but not least, studies of bilingual language acquisition should include factors that have been confirmed to influence language development in bilinguals, such as language input patterns. These criteria can be met when the study makes use of new technologies, especially mobile research applications for parents, which allow for wide-spread recruitment over large and heterogeneous samples, continuous data collection with a complex notification system to guide the users’ engagement in the study, and web-based questionnaires to provide information on additional factors relevant to the study.

New technologies naturally come with new challenges. Here, we would like to briefly recount two major challenges of using online tools and mobile applications for research: the transparency regarding the data collection, and thea high dropout rate due to the non-direct, longitudinal design of most of the online studies. As researchers, we need to ensure that the potential user is aware of what data we collect, in what way, and for what purposes. Some research applications, especially those used in health settings (mHealth apps), employ passive collection of data, i.e., without the user knowing [21]. These apps collect data from smartphone’s sensors such as GPS, Bluetooth, accelerometers, gyroscopes, and temperature sensors, as well as microphones and cameras. Data gathered with such sensors can help track disease state, response to treatment, physical activity levels, etc. Before using such an app (or any research app) the potential users must be informed, e.g., of what types of data are collected, how the collected data will be used, where it will be stored, and whether it will be shared (and if yes, how) [22,23,24]. Such informed consents, in accordance with General Data Protection Regulation (GDPR), should also be obtained from users of apps that do not passively collect any data, i.e., when the participants generate the collected information and are prompted by the app to provide particular information (active data collection [21]). Another challenge of using new technologies for research applies to longitudinal studies, and refers to the retention of the users’ engagement in the app, i.e., the number of initial users who are still actively using the app after a particular time period. The majority of mobile app users use apps over a short period of time, even when participating in a study [25,26], and some studies reported 15-day and 30-day retention rates as low as 3–4% [27]. It is therefore important to include solutions that encourage participants to actively engage in with the app for a longer period of time. Some of those solutions include gamification features and a push-notification system. Gamification refers to an inclusion of a reward system, goal setting, or game-like feedback [28] and it may involve encouraging the user to set (short or long-term) goals, awarding users when goals or usage milestones are reached. Push notifications serve as automatic reminders delivered to the user even when they are not in the app, designed to encourage the user to come back to the app [29,30]. Other challenges connected with using new technologies in research include, among others, designing the app in a user-friendly way, using US-based platforms under the European GDPR, and the lack of technical infrastructure at the university level to create and support application development. An in-depth discussion of such challenges is beyond the scope of the present paper (but see [22,23] for comprehensive discussions).

Among various new technologies, research mobile and tablet applications have been particularly compelling as the latest technological advancements were introduced in smartphones and tablets. Currently, the use of such tools in research on children is on the rise: motion sensors in tablets have enabled researchers to diagnose children with autism spectrum disorder (ASD) by assessing their motor control [31,32] and to recognize their therapy progress [33]. EasyLexia, a gamified mobile application, helps children with dyslexia improve reading comprehension, orthographic coding, short-term memory, and mathematical problem-solving [34]. Mobile applications that employ text-to-speech and voice recognition engines are used to help children learn English as a foreign language [35]. There are also commercial mobile apps that provide parents with activities promoting their child’s development and positive parenting behaviors adapted to the child’s age, such as a freely-available VROOM (www.vroom.org, accessed on 15 December 2021), or KINEDU (www.kinedu.com, accessed on 15 December 2021). However, there is little evidence yet regarding the apps’ effectiveness and reach among high-risk populations. More is known about the effectiveness of broadly defined educational apps that constitute the majority of child-targeted apps on the market, as researchers just now start to establish evaluation procedures for such apps [36]. Finally, there are a few apps developed as online language diaries for parents. BabyWordTracker App is a free mobile research app developed by Oxford Brookes BabyLab to track early vocabulary development in young children (up to 36 months), including bi- and multilingual children [37]. Parents can download the app, provide basic information on their child (e.g., age), report the child’s words (in over 14 languages) as they appear, and view the child’s progress in terms of vocabulary size. No results have been published yet on the data collected via the app.

Importantly, studies on early language have made use of indirect measures, such as parental reports, for decades. Parental reports based on standardized checklists of rigorously collected lexical items have been shown to provide reliable estimates of the child’s vocabulary size without compromising the ease of administration, which is particularly important for assessing language development of very young children. Tools such as McArthur-Bates Communicative Development Inventories (MB-CDIs, ref. [38]) have proved to be useful both in research and in early diagnosis and are now acknowledged by both academics and practitioners in a vast number of countries. Converting those pen-and-paper questionnaires into online tools and web-based apps is a practice already being adopted. DeMayo et al. [2] who introduced the Web-CDI system, report that over 3500 CDIs have been collected throughout the U.S. via their platform between 2018 and 2021. Web-based collection of CDIs is also a practice in Europe: for example, data for the Norwegian CDIs were collected through web-based online questionnaires for CDI Words and Gestures (CDI:WG), CDI Words and Sentences (CDI:WS), ref. [39], and CDI-III, ref. [40]. The online version of the CDI significantly reduces the time and expenses for data collection and coding, and reduces the risk of coding errors. Moreover, first attempts have been made to adapt the CDIs into a form of Computerized Adaptive Testing (CAT) which uses Item Response Theory (IRT) to show the parent a subset of individually selected items [41,42,43]. Such CAT CDIs can be filled in online (e.g., on a smartphone) reducing the duration of assessment while maintaining the accuracy and precision of the original tool that included the full list of items [43].

Research mobile and tablet applications are also being employed in research on early language trajectories in bilingual children. Bilingual contexts are quantitatively different from that of monolinguals: there are (at least) two languages present in the child’s environment and thus a bilingual child might typically hear less of each language when compared to the input received by a monolingual child (e.g., [44,45]). Moreover, learning more than one language in a migration context is particularly demanding due to the uneven status of the minority language (home language spoken by the family) compared to the majority language (the language of the society). As early language development lays a foundation for further developmental stages and in particular for educational performance [46,47,48], differences between monolingual and bilingual development, though subtle, may result in a relatively lower school attainment of bilingual children when attending mainstream monolingual schools in the majority language. Establishing the developmental trajectory in bilingual children would allow practitioners to accurately identify potential language delays, inform early screening practices, and eventually prevent misdiagnosis of developmental language disorder in this population.

## 2. Current Study

Our study primarily aims to establish the trajectories of early language development in monolingual, bilingual and multilingual children speaking Polish, British English or Norwegian alone, or in the combinations of Polish-British English and Polish-Norwegian. These language combinations were chosen because Norway and the UK are frequent destinations for migrating Poles. In fact, Poles were one of the largest ethnic minorities in the UK in 2021 [49] and the largest ethnic minority in Norway in 2021 [50] and Polish children in the UK and Norway constitute two large populations of Polish bilingual children in Western Europe. By collecting the data continuously (enabled by new technologies), we can collect both the children’s early gestures, words and utterances, and information regarding the children’s linguistic environment. This will allow us to directly link the quantity and quality of reported language exposure to the children’s language performance in each language (the exact timing of particular language milestones).

Our main research questions and hypotheses refer to the following issues in early language development:

### 2.1. Is the Timing of Early Language Milestones (e.g., Babbling, First Words, First Utterances) Similar in Bilingual Children’s Two Languages as Well as in Relation to Their Monolingual Peers?

With regards to the milestones in both languages of the bilinguals, previous studies (e.g., [51,52]) show that language-specific outcomes in bilingual children are dependent on various factors (e.g., input quantity) and therefore are not necessarily growing in parallel (e.g., due to unequal exposure to languages). Since we are studying bilingual children, we do not expect the children in our sample to reach all language milestones at a similar time in both their languages. With regards to the bilingual-monolingual comparison, we hypothesize that as long as we compare general language development between bilingual and monolingual children, we might expect similar outcomes between the groups, but we should also expect large variation in the data. A similar pattern was found in previous studies. Pearson et al. [51] tested vocabulary growth in bilingual and monolingual children aged 8 to 30 months and found that two measures of the bilinguals’ production in the two languages together (Total Vocabulary and Total Conceptual Vocabulary) indicated comparable vocabularies for bilingual and monolingual children. They also found large variation (in both groups) within this age span. Similar results were shown by Hoff and colleagues [44] who studied older children, aged 30 to 60 months, and found comparable vocabulary outcomes in bilingual and monolinguals when Total Vocabulary was concerned. When looking at the vocabulary in one language (English) only, however, Hoff and Ribot [52] found that the bilinguals lagged 6 months to 1 year behind the monolinguals. A more detailed description of this research question and the hypotheses can be found in the StarWords project’s preregistration: https://osf.io/pzykv.

### 2.2. What Is the Relation between the Bilingual Children’s Language-Specific Input Quantity and Quality and the Timing of Reaching Particular Language Milestones (e.g., Babbling, First Words, First Utterances) in That Language?

With regards to input quantity, we will investigate whether the amount of time (during the day) that the child is hearing each language will impact the timing of reaching particular language milestones. Pearson et al. [51] found a strong positive correlation (*r* = 0.82) between language-specific input and vocabulary growth in bilingual children aged 8 to 30 months. The same relation between input and language outcomes might be possible for the timing of reaching early language milestones in our bilingual samples. With regards to the input quality, we will use frequency of book reading treated as a proxy of input quality, however, if the data allow, in the exploratory analyses we will include other input quality measures, i.e., play time, singing, TV watching, media use, and the number of speakers using L1 and L2 towards the child. We hypothesize that the frequency and length of language-promoting activities will impact the timing of reaching particular language milestones. Similar results were shown in previous studies. Patterson [53] found that the frequency of book-reading in each language of bilinguals was related to expressive vocabulary size in the same language, and the relationship was significant even after taking overall input quantity into account. Building on these results, Scheele et al. [54] found that 3-year-old bilingual children’s rate of vocabulary growth was strongly influenced by the parents’ language use in oral and literate activities (including frequency of reading and storytelling). In a recent study, Kartushina et al. [7] have identified book reading as the major positive contributor of expressive vocabulary size in monolingual children aged 8–36 months. Frequency of book reading had more positive impact on children’s expressive vocabulary than any other examined activity (e.g., singing, TV watching). A more detailed description of this research question and the hypotheses can be found in the project’s preregistration: https://osf.io/pzykv.

### 2.3. What Are the Relations between Sleep Patterns, Breastfeeding and Early Language Development?

We will investigate whether sleep patterns (consecutive daytime sleeping durations, consecutive nighttime sleeping durations, ratio of consecutive daytime sleeping durations to consecutive nighttime sleeping durations, total length of sleeping) and breastfeeding patterns (whether the child is breastfed at a given age, total length of breastfeeding) are related to the very early language development (emergence of the babbling, earliest words and multiword utterances, CDI scores) in monolingual and bilingual children. Generally, breastfeeding is found to have a positive impact on language development, especially in populations under the risk of developing language impairment (see [55] for review). The length of breastfeeding is also positively correlated with the likelihood of polysyllabic babbling at the age of 8 months [56], as well as with expressive and receptive communication scores at the age of 18 months [57]. Another study showed that even breastfeeding for more than 12 months was beneficial for toddlers’ language development [58]. We thus hypothesize that breastfeeding will be positively related to the children’s CDI scores at 2 and 3 years. We also hypothesize that children breastfed at the age of 3/6 months will start babbling at a younger age than children who are not breastfed at the same age, while maternal education, child birth weight, and child gender are controlled. Regarding the sleep patterns, the day/night sleep duration ratio which is naturally decreasing with child development (i.e., in development children tend to sleep more at night and less during day) may be a good index of sleep maturation [59]. It was shown that the day/night sleep duration ratio at the age of 6 and 18 months (but not 30 months) is weakly related to the language outcomes (CDI scores) at the age of 18 and 30 months, and to receptive vocabulary scores at the age of 60 months [59]. Dearing et al. [60] also found a positive influence of sleep patterns on language development (comprehension and production). In addition, sleep patterns and breastfeeding are not independent of each other. Breastfed children are more likely to wake up during the night [61], although the total number of slept hours does not necessarily differ between breastfed and non-breastfed infants [62]. It appears that the relations between breastfeeding, sleep patterns, and language development may not be clear: whereas both breastfeeding and longer sleep increase vocabulary scores, breastfeeding may decrease the sleep duration. A more detailed description of this research question and the hypotheses can be found in the project’s preregistration: https://osf.io/rb9g7.

### 2.4. Is There a Clearly Observable Vocabulary Spurt in Monolingual and Bilingual Children?

Vocabulary spurt, i.e., a transition to rapid word-learning [63], is one of the developmental changes found in many children (but not all, cf. [64]). It is suggested that in order to build extensive vocabularies, children cannot employ a steady rate of learning, but at some point (typically in their second year of life) have to show an accelerated rate of word-learning. Researchers have used many different methods to operationalize this vocabulary spurt but Ganger & Brent [64] suggest that it should be identified as a transition between a slow word learning stage and a faster word learning stage (and not a gradually increasing learning rate) and that the faster word learning stage should be sustained for some period of time. Having operationalized the vocabulary spurt similarly as Ganger & Brent [64]—as a transition between a slow and a sustained faster learning stage, we hypothesize to find a minority of children with a clear vocabulary spurt, and that the proportion of children showing/not showing a vocabulary spurt will be similar in the monolingual and bilingual group. A more detailed description of this research question and the hypotheses can be found in the project’s preregistration: https://osf.io/ap94y. 

## 3. Methods

The StarWords study is an observational study and the subjects are not assigned to any treatment. Below, we are describing the sample and eligibility criteria, our recruitment procedures, the exclusion criteria for the future analyses and the data collection procedures.

### 3.1. Sample and Eligibility Criteria

The study is on-going and the recruitment started in mid-June 2021. Our recruitment is targeted primarily at parents of children exposed to Polish and British English (living in the UK), parents of children exposed to Polish and Norwegian (living in Norway) and parents of children exposed to Polish (living in Poland) aged 0–24 months. Additionally, we also aim to recruit parents of Norwegian monolingual and British monolingual children. While we recruit children in their first and second year, we encourage parents to be active in the app when the child is older than 2 years. The Ethics Committee at the Faculty of Psychology, University of Warsaw approved the StarWords project’s procedure (including recruitment and data collection) and the Data Protection Services in Norway has assessed that the processing of personal data in this project is in accordance with data protection legislation. Our sample size is estimated according to the potential availability of participants: Our aim is to gather longitudinal data from altogether 200 bilingual participants (100 Polish-Norwegian, 100 Polish-British English) and 200 monolingual participants (100 Polish, 100 Norwegian). Currently (March 2022) there are almost 4000 parents (who entered the study within the children’s age range, i.e., 0–24 months) using the app. This includes more than 20 Polish-Norwegian bilingual children, 90 Polish-British English bilingual children, and 2000 Polish monolingual children. Although the current monolingual Polish sample exceeds our aim regarding the sample size, we expect that many of the parents may use the app for a relatively short period of time and thus for some longitudinal analyses the final sample may be substantially smaller. In order to achieve the expected sample sizes, we are recruiting continually, i.e., parents are invited to join at any point of the study, provided they meet our eligibility criteria: They are parents of Polish-speaking bilingual or multilingual children or Polish, Norwegian, or UK-English speaking monolingual children aged 0 to 24 months. A bigger final sample would certainly make the subsequent analyses more powerful, thus we will not stop recruitment even if the minimal numbers per group are achieved and secured for a longer period of time.

Though the focus of the study is on Polish-speaking children living in Poland, Norway and the UK, the StarWords app is available publicly in Google and Apple app stores, and has drawn considerable attention from Poles living in Poland who intentionally raise their children bi- and multilingually (“elective” or “intentional bilingualism”). Currently (March 2022) parents of over 250 such children are using the app. Moreover, many parents using the app are Poles living in other countries than those of our original interest and speaking other language combinations than Polish-English and Polish-Norwegian. Currently (March 2022) parents of 160 such children are using the app. These families live all around Europe, e.g., in Germany (60), France (14), the Netherlands (13), Switzerland (12), Denmark (11), Spain (11), Belgium (10) but also USA (9), New Zealand (2), South Korea (2), Iceland (1). If the data allow, we consider exploratory analyses with these samples.

### 3.2. Recruitment

Our recruitment is continuous, i.e., ongoing through all of the time of the study (ca. till spring 2023). A large share of our recruitment includes online procedures. We are recruiting actively via social media, i.e., Facebook and Instagram posts and short-term advertisements. We are contacting—via social media and email—public figures known for their interest in research and child development, i.e., bloggers, influencers, practitioners, and researchers actively involved in research communication practices. We are also contacting university media outlets to spread the information about the project. Finally, we have built the project’s webpage (http://starwords.eu/) to create a space on the internet where parents can learn more details about the study, and have set up Facebook and Instagram channels for the research team (FB: @multilada.uw, IG: @multilada_uw) to enable parents to easily contact us. Importantly, parents receive detailed information on the study prior to their decision on participation: either via the website or (if they find the app in the stores without visiting the study’s webpage) directly in the app, before registering into the study.

### 3.3. Exclusion Criteria

Before analyzing the data, we will exclude from the sample children with confirmed health problems (i.e., temporary hearing loss, frequent ear infections, children born prematurely or children whose birth weight was smaller than 2.5 kg). In our main analyses of bilingual data, we will also exclude children who are bilingual with Polish and Norwegian/English but neither of their parents is a native speaker of English/Norwegian and the family is living in Poland (i.e., intentional bilingualism as opposed to bilingualism in the context of migration, which is the focus of the present study). However, if the data allow, we would like to plan some exploratory analyses on this group as well.

### 3.4. Data Collection

**Figure 1:**
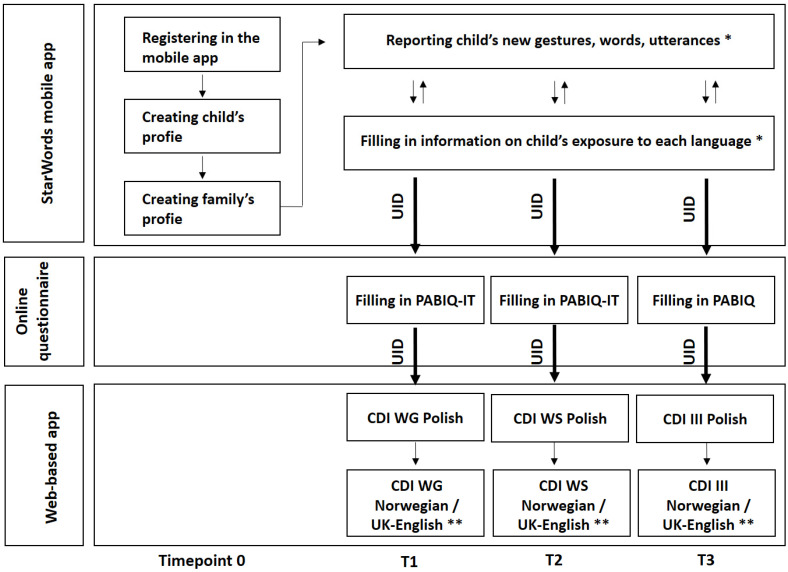
Study procedure. The data collected with the three tools (StarWords mobile app, online PABIQ [-IT] questionnaires and web-based CDIs) is matched via the traveling unique user identifier (UID) not overtly known to the participant (see Section 3.4.4). Note. * continuous data collection (see Section 3.4.1). ** Language dependent on the type of bilingual group (Polish-Norwegian or Polish-British English). Polish monolinguals receive CDIs in Polish only (see Section 3.4.3).

The data is gathered from three sources: the StarWords mobile application (see Section 3.4.1), an online modified version of the parental questionnaire PABIQ (-IT) (see Section 3.4.2), and the web-based CDIs (see Section 3.4.3). Figure 1 presents the general study procedure and the connection between the study tools.

Before joining the study, the parents are informed about the app’s research purpose, the purpose of the notifications, their ongoing access to the entered data and the study procedure. They are also informed that the study is confidential, that the collected data will be used for scientific purposes only and presented collectively. The information they receive on their personal data collection (i.e., the child’s name, the parent’s email address) is compliant with the GDPR. The parents are informed that the collected data is stored on secured servers located in the European Union, and will not be distributed to any third parties (outside of the research team), and that they can withdraw from the study at any point without giving a reason.

The data is gathered (partly) longitudinally. Parents are asked to download the “StarWords—every word counts” app, and report on their child’s new gestures, words and utterances as they appear, and answer questions e.g., about the child ’s language environment (more details in Section 3.4.1). At a specific age, parents are asked to fill in a language background questionnaire (see Section 3.4.2) and MacArthur Bates Communicative Development Inventories (CDIs) questionnaires (see Section 3.4.3) in Polish and/or Norwegian or British English. Parents entering the study are encouraged to stay as long as possible, but are allowed to leave the study at any point without giving any reason and if they want their data removed from the study’s database, they can require this via the app or email.

Parts of the data are gathered cross-sectionally. Specifically, some participants stay in the study for a relatively shorter time (e.g., they opt out early, or may join in towards the end of data collection). These data will be used for particular analyses to enhance their power.

#### 3.4.1. The “StarWords—Every Word Counts” Mobile Application

We have created a mobile application specifically for the purpose of the present study, “StarWords—every word counts”, henceforth referred to as the StarWords app. Creation of the app was a complex and long process, and we aim to eventually open the source code for modification so that our work can be beneficial for other researchers interested in using such an app in their research. The StarWords app has a twofold purpose: first, it serves as an online language diary in which parents and/or guardians are encouraged to enter early gestures, words, neologisms and utterances their children produce in real time or shortly after the production. Parents can also choose to add other guardians to their family account (e.g., the other parent, grandparents, nanny, or teacher), who will also download the app and report children’s gestures, words and utterances. Importantly, parents can see all the information reported by other guardians in the app, while other guardians cannot see the information entered by the parents. Second, the app allows us to collect information on the children’s background characteristics (e.g., country of residence, languages used with and by the child, the presence of health problems), linguistic environment (the quantity and quality of exposure to each language, the age of first contact with languages), the child’s early development (e.g., age of crawling, walking unassisted, and babbling), and some other child and family characteristics (e.g., the child’s sleep patterns, the number of siblings, and parental education and language use).

The sampling of the data is continuous and largely dependent on the user, i.e., the parent may report the child’s early gestures, words and utterances as they appear, or as they find the time and energy to do so. However, the app also includes a complex push notification system to promote the parents’ retention in the study. Depending on the child’s age and the parent’s current engagement in the app, the parents receive various prompts: if the child is at an age when we expect some first words to appear (i.e., 10–12 months), users are prompted to reflect on whether the child uses some gestures or repeats some words during everyday routines (e.g., “Think for a moment, does [child’s name] mimic various sounds, e.g., made by animals, vehicles, etc.?”). If the child is already speaking and some items (e.g., words, utterances) have been reported, but the user has not been active for two weeks, users are prompted to think about the words the child uses in various everyday situations, e.g., when eating, bathing, going outside (e.g., “Think for a moment, what does [child’s name] do when you’re singing together or you’re reading/watching picture-books together? Does [child’s name] try to repeat after you or try to talk?”). Once every 9 days, parents of bilingual children are prompted to report on the child’s exposure to each language on the day before. Since the question is asked every 9 days, some measurements will refer to week days, some to weekend days, and altogether parents will be asked about the language exposure twice a month. If some family’s characteristics are not given by the parent, the parent is prompted once a month to answer questions in that respect.

All the data is collected actively, i.e., when the users provide the information either spontaneously or in response to notifications [21]. No passive data collection is used in this study, i.e., we do not collect data from the device’s sensors, such as GPS, Bluetooth, microphone, or cameras. However, parents are given the opportunity to upload audio recordings of how children pronounce particular words (this is not obligatory or required of them). As mentioned in the introduction, one of the challenges of non-direct longitudinal testing, e.g., with the use of a mobile app, is the retention of the participants over time. Our strategies to support retention include: a user-friendly and interesting design of the app, reports and summaries of data entered by the parents, access to podcasts with researchers studying child bilingualism and personalized coloring booklets (in .pdf format). Importantly, access to these materials is not given on the basis of the number of items (i.e., gestures, words, and utterances) the user reports, as this could push parents toward over-reporting, as well as cause increased pressure on families with children who are late talkers or potentially may have developmental language disorder. Instead, access to some of the materials is awarded when the parents respond to the push notifications (e.g., fill in their basic family characteristics, answer regular questions about language environment, fill in a CDI).

#### 3.4.2. PABIQ and PABIQ-IT

In our attempt to move all the testing online, we have also created a modified online version of the parental questionnaire PABIQ-IT (for infants and toddlers), “Kwestionariusz dla rodziców dzieci jedno-i wielojęzycznych: niemowlęta i dzieci najmłodsze”, [65] (original PABIQ-IT, Parents of Bilingual Children Questionnaire—Infants and Toddlers: [66]) and PABIQ for children aged above 3, “Kwestionariusz dla rodziców dzieci jedno-i wielojęzycznych: dzieci przedszkolne i starsze” [67] (original PABIQ, Parents of Bilingual Children Questionnaire: [68]). The PABIQ and PABIQ-IT questionnaires collect information about the child’s language environment (e.g., number of languages used towards the child, frequency of use, contexts in which each language is used, and the child’s skills as reported by parents), and general information about the child and family (e.g., date of birth, early development, parental education, and difficulties at school experienced by family members). The data from the questionnaires are collected together with the CDIs at three timepoints: when the child is between the ages of 16–18 months (PABIQ-IT), 19–26 months (PABIQ-IT) and when the child is 36 months old (PABIQ) (Please note that we recruit children aged 0–24 months and as the study is longitudinal, children entering the study in their second year of life may still be participating in the study in their third year of life). These questionnaires are relatively long (up to 12 pages, depending on the language version and the formatting). Converting these paper questionnaires into online forms allowed us to add a system of conditional logic, according to which certain questions (e.g., those regarding bilingual setting) can be hidden from a monolingual parent. Thus, moving the PABIQ-IT and PABIQ online allowed us to significantly shorten the time needed to fill in the questionnaire.

#### 3.4.3. CDIs

MacArthur-Bates Communicative Development Inventories (CDIs) are standardized parental questionnaires designed to measure language development of young children [25]. They have been adapted to many languages and are widely used in various countries for both research and diagnostic purposes. The parent’s task is to mark words (from a list of rigorously collected lexical items) that their child understands and/or uses. There are three age-versions of the CDIs: Words and Gestures (CDI:WG) for children aged from below 1 to about 2 years, Words and Sentences (CDI:WS) for children aged 2–3 years, and CDI-III for children beyond 3 years old. The exact age ranges as well as some other details differ across adaptations but in general CDI:WG asks parents for comprehension and production separately and there is a list of gestures, routines etc. along the word checklist, whereas in the versions for older children parents are asked for the production only and there are some questions about grammar in addition to the word checklist. In the StarWords project we use existing adaptations to Polish [69], Norwegian [40,70], and British English [71,72]. Part of the project is also to develop Polish CDI-III.

The data from the CDIs will be collected at three timepoints: we first ask parents to fill in CDI:WG at the age of 16 months, then at the age of 26 months we ask them to fill in CDI:WS, and we ask them to fill in CDI-III when their child is 36 months old (see Figure 1). Because of the continuous recruitment, children may be of any age (between 0 and 24 months) at the time their parents register in the app. Therefore, if they are between 17 and 24 months old, we also ask their parents to fill in a CDI when they enter the study (on the next day after their registration): up to the age of 18 months they are given CDI:WG, between 19 and 24 months of age they are given CDI:WS, and if the child is between 22 and 24 months old they are not asked to fill in CDI:WS again at the age of 26 months. If the only language registered for the child in the mobile app is Polish, parents are asked to fill in only Polish CDIs, whereas if the child’s languages registered in the app are Polish and Norwegian or British English they are also asked to fill in Norwegian or UK CDIs (if the parent does not feel comfortable in filling in a CDI in the child’s second language, they can forward it to another parent, care-taker or teacher. For some CDIs we plan to gather both full-list data and Computer Adaptive Testing (CAT) measurements and we will use the study to validate the CAT approach to CDIs [42]. More specifically, participants will be invited to fill in both the full-list Polish CDI:WG and CDI:WS and their CAT versions, in which the next item shown to the user is chosen on the basis of the users’ previous responses. Such dual testing will allow us to check whether the scores of the full-list and CAT version are related, which would inform us of the CAT version’s validity. 

We have developed a web application, CDI-Online, for online administration and data collection of CDIs. The app is integrated with the rest of the project, and with the mobile StarWords app in particular: the mobile app generates a personalized URL for parents to access the relevant CDI version at a particular timepoint, notifies parents if they have not filled it in (every three days but no more than three notifications), provides general feedback and a small reward on completion, and, if applicable, seven days after the completion or after the last notification it generates a URL to access a next CDI (e.g., Norwegian or British English or a CAT version).

Importantly, CDI-Online is fully configurable and we intend to provide its code as open-source to researchers interested in gathering CDI data online. To use the app, one needs a web server configured to run software written in R and Shiny [73,74] and a MySQL database set up. All configuration is done via a small set of text files, containing items, instructions and various settings. If percentile norms are provided as well, the app can be configured to check raw scores against the norms and act accordingly. Crucially, the app can run CDIs in a CAT mode if provided with relevant IRT estimates for items (i.e., individual items’ difficulty and discrimination, i.e., how well a given item differentiates between ability levels), and stopping criteria. It can also be set up to gather data on several age and language versions. CDIs can be accessed from a PC or a mobile device via a URL containing as parameters language and age version, the type of measurement (CAT or full-list), and a unique ID. The same URL may be used to resume filling in the inventory at a later date on the same or different device. All responses are securely stored in the MySQL database.

#### 3.4.4. Data Safety

Altogether, three types of tools are used in the StarWords study: a mobile app (StarWords), a web-based application (CDIs), and online questionnaires on a web-page (PABIQ-IT and PABIQ). Importantly, the data gathered from the three separate tools can be matched to a particular participant via a unique user identifier (UID), not overtly known to the participant (see Figure 1). The UID is a 21-character-long code, containing both letters (lower and upper case) and special characters. It is generated automatically within the StarWords app, pseudo-anonymized, and carried over within the url address to the other tools in the study (e.g., the PABIQ-IT questionnaire or CDI). The UID allows us to match the data between the tools, without asking the participant to provide or copy and paste an identifier, and without using the participant’s personal data (e.g., name, e-mail address).

The data collected in the study via the StarWords app and the CDIs are stored in a MySQL database on a Virtual Private Server (VPS) located in the EU and hosted by an external company, with whom the University of Warsaw has signed a data processing agreement (OVH). Access to the VPS is only provided to authorized users with whitelisted IP and necessary credentials. The data from the online questionnaires, i.e., PABIQ-IT and PABIQ, are stored online with no personal data and just the UID. The users of the StarWords app are allowed to delete their account and data at any time. As they delete their data from the app, this data will also be removed from the MySQL database.

## 4. Outcome Analyses

The study is on-going and no analyses have been performed on partial data. The start of the recruitment and data collection was in mid-June 2021, and data collection will last approximately till spring 2023. A detailed description of the outcome variables and planned statistical analyses can be found in the project’s pre-registrations: https://osf.io/3ruj9/registrations. 

With regard to earlier stated hypotheses and research questions we plan on performing the following analyses.

Re 1.: To check for the timing of early language milestones, we plan a between-subjects design to compare the age (in months) of reaching particular linguistic milestones across bilinguals and monolinguals. We will also perform a within-subjects design to compare the timing of reaching particular linguistic milestones in bilinguals’ first (L1) and second language (L2).

Re 2.: We plan to perform a series of correlational analyses of the quantity and quality of language input and the timing of reaching particular linguistic milestones in bilinguals’ L1 and L2. As a proxy of input quantity, we will use the amount of time (during the day) that the child is hearing each language, and as a proxy of input quality, we will use frequency of book reading, however, if the data allow, in the exploratory analyses we will include other input quality measures, i.e., play time, singing, TV watching, media use, the number of speakers using L1 and L2 towards the child. 

Re 3.: In order to study the relations between sleep patterns, breastfeeding and early language development, we will perform a series of regression analyses for monolinguals and bilinguals. We will use language measures as the dependent variables. The independent variables will include breastfeeding length in months, the total length of sleep at various ages (in months), the day/night sleep ratio at various ages (in months).

Re 4.: With regard to the vocabulary spurt in mono- and bilingual children the analysis will consist of three steps: (1) incorporating Ganger & Brent’s [64] procedure to determine the ratio of children showing the trend of faster word learning stage; Ganger & Brent (2004) define vocabulary spurt as a transition between a slow word learning stage and a faster word learning stage (and not a gradually increasing learning rate) with the faster word learning stage sustained over time; this is best described by a quadratic function, and we will determine the number of children whose rate of word learning over time fits a quadratic function; (2) comparing the ratio of spurters across the two studies groups: bilinguals and monolinguals, and afterwards (3) for the spurters, we will find the mean age and the mean vocabulary size of reaching the inflection point (the transition between the slow and fast word-learning rate). We will compare the vocabulary size and the age of reaching the vocabulary spurt between monolingual and bilingual spurters.

With regard to potential exploratory analyses, we consider comparisons of the sample of bilingual children raised in the migration context (as described above) with the sample of bilingual children experiencing elective/intentional bilingualism (living in Poland) and also bilingual children in the migration context acquiring other languages than English or Norwegian (beside Polish).

## 5. Discussion

We have presented the new, on-going study “StarWords: a study on parental report on words” which aims to investigate early language development trajectories in mono- and bilingual children using mobile and web-based applications. These allow us to reach a large and linguistically heterogeneous sample (without the cost of sending out paper questionnaires) and gather data longitudinally and continuously. Although our primary focus is on Polish- British English and Polish-Norwegian bilinguals of migrant background (and respective monolingual groups), with the use of mobile and web-based applications, we are able to reach linguistically heterogeneous samples of bilingual and multilingual families living all across the world. The app is freely available via Google Play and Apple App stores, and we are observing an influx of families speaking various language combinations using the app. Since we have obtained Ethics clearance to include in our study parents of children who are outside our original scope (Polish and Norwegian monolinguals, Polish- British English bilinguals and Polish-Norwegian bilinguals), but have registered in the study, we are now collecting this data with an aim of exploring language trajectories across culturally and linguistically diverse populations of bilingual and multilingual children.

Further, these apps allow us to gather information on a particular child’s language development from many sources: not only parents, but also other care-takers that parents may wish to add, e.g., grandparents, nannies, and teachers. This solution is particularly important in the bilingual context, e.g., when the parent does not feel comfortable in reporting the child’s progress in L2, and a nanny or a teacher might help. The parent can invite the other care-taker to download the app (via invitation sent from the app) and this care-taker can then report the child’s language progress in their account. Finally, we can include factors that are assumed to influence language development in bilinguals, such as language input patterns. As a result, we hope to establish the exact timing of achieving particular language milestones and observe how language-specific input quantity and quality affects the timing of reaching particular language milestones in bilingual children. We believe that these goals can be achieved by the use of web- and mobile applications for parents with a complex notification system to guide the users’ engagement in the study, and online questionnaires to provide information on additional factors relevant to the study.

When designing the StarWords study and the online tools, we were naturally compelled by the advantages of the online tools: with wide-spread smartphone coverage we expected to be able to reach a larger and a more diverse set of participants. However, there are two sides to every coin. As Lourenco & Tasimi [75] point out, with the COVID-19 and new challenges on family care while working from home, the participants in online studies may now largely comprise those who have the time and resources to participate in such studies. On the plus side, with online testing there is no need for direct contact with the study participants, which decreases the research assistant and traveling costs. However, the effort and costs of designing successful and user-friendly apps and their on-going technical support can also be substantial. Sometimes the university technical infrastructure may not be thoroughly prepared to provide proper support and help in designing and deploying online tools. The continuous data collection means that parents can report the first gestures, words and utterances as they appear, and therefore we do not depend on their ability to recall or remember their children’s first words. On the other hand, we have little control over what the parents report, and of their frequency of reporting and of their engagement in the study. The complex notification system, gamification and rewards become crucial and largely invaluable in supporting user retention, but they must not be overdone: we do not want to overwhelm the parents/users with too many notifications, and the notifications have to be tailored to different potential cases (e.g., if a child is a late-talker, too many notifications asking about the child’s new words might be a source of additional worry to the parent). The gamification and rewards can prolong the parents/users’ engagement in the app, but they should not change the parental behaviors towards their children in an observation study, and we should reward the users’ engagement in the app, instead of rewarding reporting new words, which could lead to falsified data or frustration in parents of late-talkers. Additionally, the rewards need to be digital, e.g., podcasts or articles, to rule out shipping costs.

One of the challenges of the online testing is also understanding and being able to explain the data collection and storage procedures to the study participants and authorities. This includes informing the participants about the study procedure, its purpose, and the participants’ access to the entered data. We also inform our participants about their rights following from GDPR. Using online tools and international collaboration lengthens the process of obtaining ethical approvals, as many additional details are required concerning data storage and processing, technological solutions employed when designing the tools and the databases. On a positive note, such procedures make us alert and conscious of the need to make the data collection and storage procedures in accordance with current law as well as becoming transparent to the study participants. Moreover, in the effort to solve the logistic complexity of merging data from different online tools while keeping with the ethical guidelines, we were able to find new solutions, such as the unique user identifier (UID) codes, not overtly known to the participant. This also implies less communication of personal data and smaller risks of data security breaches, when compared to assigning the participants overt id codes. The UID allows us to match the data between the tools, without asking the participant to provide or copy and paste an identifier, and without using the participant’s personal data (e.g., name or e-mail address).

Finally, in the perspective of the COVID-19 pandemic, mobile technologies enable testing with no direct, personal contact. As a result, more and more research teams are developing online tools for research, despite the new challenges linked to online testing, showing that new times call for new measures. Not only that, but researchers can join in efforts to establish unified, discipline-wide platforms for supporting large-scale research projects. Some such platforms existed well before the pandemic, e.g., Child Language Data Exchange System (CHILDES, ref. [76]). However, one such new attempt is the Collaboration for Reproducible and Distributed Large-Scale Experiments (CRADLE) [77], a shared infrastructure for recruitment, data collection, and data sharing across many universities and countries. Its novelty lies in the fact that potential participants registered in CRADLE could be invited to take part in many different studies that match their background characteristics.

However, COVID-19 may ironically provide new limitations on the online testing. As Lourenco & Tasimi [75] point out, more and more people lose their jobs due to the difficulties associated with the pandemic, and internet access may become a luxury. Moreover, public access to the internet may also be restricted due to lockdown and/or local restrictions. In our StarWords study, we have included a safeguard against that by allowing offline reporting and automatic synchronization of data once the phone is connected to the internet.

All in all, the use of new technologies in social sciences, and especially in developmental studies, is, as Sheskin et al. [77] put it, an “overdue acceleration”. Even as new challenges arise, researchers are still motivated to develop new solutions and learn new skills in order to be able to design new tools for online testing. We strongly believe that as researchers re-evaluate their research infrastructure to support online testing, their expansion into the online world will help to push the current limits of the field.

## 6. Conclusions

In this study protocol we have presented the new, on-going study “StarWords: a study on parental report on words” which aims to investigate early language development trajectories in mono- and bilingual children using mobile and web-based applications. With the use of mobile and web-based applications, we are able to reach linguistically heterogeneous samples of bilingual and multilingual families living all across the world. These tools allow parents to report their children’s language development as it progresses, while allowing us to characterize children’s performance in each language (the age of reaching particular language milestones). Establishing the developmental trajectory in bilingual and multilingual children would allow practitioners to accurately identify potential language delays, inform early screening practices, and eventually prevent misdiagnosis of developmental language disorder in this population.
